# Preparation of mucosal nanoparticles and polymer-based inactivated vaccine for Newcastle disease and H9N2 AI viruses

**DOI:** 10.14202/vetworld.2017.187-193

**Published:** 2017-02-14

**Authors:** Heba M. El Naggar, Mohamed Sayed Madkour, Hussein Ali Hussein

**Affiliations:** 1 Department of Poultry Vaccines Production Unit Veterinary Serum and Vaccine Research Institute, Abbasia 11759, Egypt; 2 Department of Virology, Faculty of Veterinary Medicine, Cairo University, Giza 12211, Egypt

**Keywords:** adjuvant, H9N2, mucosal, nanoparticles, Newcastle, polymer

## Abstract

**Aim:**

To develop a mucosal inactivated vaccines for Newcastle disease (ND) and H9N2 viruses to protect against these viruses at sites of infections through mucosal immunity.

**Materials and Methods:**

In this study, we prepared two new formulations for mucosal bivalent inactivated vaccine formulations for Newcastle and Avian Influenza (H9N2) based on the use of nanoparticles and polymer adjuvants. The prepared vaccines were delivered via intranasal and spray routes of administration in specific pathogen-free chickens. Cell-mediated and humoral immune response was measured as well as challenge trial was carried out. In addition, ISA71 water in oil was also evaluated.

**Results:**

Our results showed that the use of spray route as vaccination delivery method of polymer and nanoparticles Montanide™ adjuvants revealed that it enhanced the cell mediated immune response as indicated by phagocytic activity, gamma interferon and interleukin 6 responses and induced protection against challenge with Newcastle and Avian Influenza (H9N2) viruses.

**Conclusion:**

The results of this study demonstrate the potentiality of polymer compared to nanoparticles adjuvantes when used via spray route. Mass application of such vaccines will add value to improve the vaccination strategies against ND virus and Avian influenza viruses.

## Introduction

Avian influenza virus (AIV) subtype H9N2 is low pathogenic avian influenza virus and it causes serious economic losses in poultry industry. The H9N2 subtype outbreaks occurred in Germany during 1995 and 1998 in ducks and turkey, Italy in 1994 and 1996 detected in chickens, Ireland in 1997 also isolates from pheasant, ostriches in South Africa in 1995, turkeys in the USA between 1995, and 1996 and finally in chickens flocks in Korea 1996 [1-3].

In 1997, H9N2 virus outbreaks distributed in Asian countries such as Saudi Arabia, Iran, China, Pakistan, and other countries [4].

In Egypt [5], the presence of H6 and H9 AI antibodies (Ab) among broiler and layer breeders flocks were detected using serological tests such as ELISA and hemagglutination inhibition (HI) tests. In 2003, the H9N2 samples have been collected from live bird markets then characterized in the USA by the help of NAMRU [6]. The first publication for isolation of H9N2 from Egypt was at 2011 which isolated from commercial bobwhite quail [7]. The hemagglutinin gene sequence of the isolated Egyptian viruses showed the highest similarity with one of the recent Israeli strains (97%) detected from 2006 to 2010 [6]. Now H9N2 subtype AIVs vaccination has been used to face the field outbreaks [8].

Newcastle disease virus (NDV) is one of the most devastating diseases of the poultry industry which shows genetic diversity and complexity [9]. NDV was discovered in 1926 and since that time the NDV was classified to be one serotype and to date can be divided into Class I (9 genotypes) and Class II (15 genotype). The new virulent genotypes were observed through the detection of the genomic sequence changes of NDV of low and high virulence at different geographic locations across the world [10-12]. Velogenic NDV caused economic losses among commercial chicken in Egypt [13]. Researchers in vaccines production gave more concerns to develop vaccines that induce protective immunity to prevent and control the virus evolution in poultry flocks [14]. The antigenic diversity of the NDVs isolated from 52 breeders and broiler flocks in Egypt [15], and the genetic resistance of the NDVs in the Egyptian native breeds has been reported [16]. The virulence determined using sequence gene analysis was carried out for local velogenic isolate SR/76 [17]. NDV genotype VII was isolated from H5N1 infected broiler flock 2012, this isolate was closely related to Chinese strains so this study reports the characterization of NDV genotype VII in broiler chicken with co-infection with Avian influenza H5N1 virus [18]. The Chinese VII d virus with sever outbreaks of ND was characterized by Radwan *et al*. [13].

The delivery of inactivated antigens by injection may enhance the humeral immune response but rarely induce mucosal immune response. In the other side, the mucosal antigen delivery can enhance both local and systemic immune responses, which provides an advantageous approach for immunization [19]. The delivery of inactivated influenza virus was tested in mice and resulted in protection against different viruses [20-24]. Mucosal vaccination strategy could be rapid and preventive method of vaccination in outbreaks in endemic areas and it should be suitable for mass application even applied by spray or aerosole and effective after single application [25-29]. In normal circumstances, the inhaled antigen do not trigger strong immune responses through contacting with the respiratory tract mucosa but induce a state of tolerance [30,31] leading to a tolerogenic environment in the mucosa. The applied whole inactivated virus by intranasal route alone is poorly immunogenic [31-33]. To enhance the immunogenicity of whole inactivated virus, it needs to be adjuvanted either with Montanide™ Gel 01 ST (Gel 01) which is a polymer based adjuvant (gel particles of sodium polyacrylate in water) or Montanide™ IMS 1313 N VG (IMS 1313 N) (nanoparticles in an aqueous phase containing an immunostimulating compound). Both are suitable for mass vaccination that can be used in intensive poultry industry via spray, shower or drinking water [34-36]. In this study, we prepared two inactivated vaccines based on the use of such adjuvants, and comparative study of the immune response as well as challenge trial was carried out.

## Materials and Methods

### Ethical approval

All applicable institutional guidelines for the care and use of animals were followed.

### Antigen

Egyptian two local isolates including influenza A virus (A/chicken/Egypt/114922v/2011(H9N2), and NDV (NDV-B7-RLQP-CH-EG-12) virulent were kindly provided by National Laboratory for Veterinary Control on Poultry Production, Animal Health Research Institute, Egypt. Sequences of these viruses have been found in gene bank under the following accession numbers: for AIV (JQ419502) (sequence of HA gene) and for NDV (112RKQKR*F117KM288609) (sequence of F gene).

### Adjuvants

Montanide™ IMS 1313 N VG (IMS 1313 N) and Montanide™ Gel 01 (Gel 01) and as well as Montanide™ ISA 71 VG were used in this study. Montanide™ IMS is ready to dilute range of adjuvants consisting of liquid particles (10-500 nm) dispersed in an aqueous phase containing an immunostimulating compound. Montanide™ Gel 01 is ready to dilute polymeric adjuvant. It contains gel particles of sodium polyacrylate in water. Montanide™ ISA 71 VG It is a mineral oil based adjuvant that has been developed for manufacture of water-in-oil (W/O) emulsion. The three adjuvants were kindly provided by SEPPIC Co.

### Vaccine formulation

IMS 1313N nanoparticles adjuvant was used in ratio (weight) 50% in aqouse phase; whereas Gel 01 Polymer adjuvant was used in a ratio 10% with formulation process recommended by manufacture. W/O emulsion vaccine using Montanide™ ISA 71 VG at a ratio of 30/70 (v/v) aqueous/oil ratio was also prepared. Stable preparations are obtained by mixing the aqueous medium into the Montanide™ ISA 71 VG, at room temperature or less, under vigorous stirring (for 15-30 min) as recommended by manufacture the antigen content in all the prepared vaccines not <10^8.5^ embryo infective dose 50% (EID_50_).

### Challenge trial

About 300 specific pathogen-free 10 days old chickens are divided into six groups (50 chickens each), Groups 1 and 2 were vaccinated with bivalent NDV and H9N2 inactivated vaccine adjuvanted with IMS1313. The vaccine was delivered by intranasal route in Group 1 (each animal received 1 antigen dose in 0.1 ml injected evenly in both nostrils) and spray route in Group 2 (2.5 ml of solution containing 10 doses of vaccine were sprayed over 10 birds in a box). Groups 3 and 4 were vaccinated in dose similar to Groups 1 and 2 with bivalent NDV and H9N2 inactivated vaccine adjuvanted with GEL 01. Group 3 vaccinated by intranasal and group 4 by spray routes. Group 5 was injected I/M with 0.5 ml of inactivated bivalent NDV and H9 adjuvanted with Montanide ISA 71, and finally, Group 6 were kept non-vaccinated chickens as control negative group. After 3 weeks, postvaccination 10 birds from each of Groups 1 to 5 were challenged with 10^6^ EID_50_ NDV via ocular rout and another 10 birds from each group were challenged with 10^6^ EID50 H9N2 virus also, 20 birds from Group 6 were challenged in separate isolates with NDV and H9N2 (10 birds each) and kept as positive control for the challenged viruses.

Heparinized blood samples from chicken in the six groups at the 1^st^, 3^rd^, 5^th^, 7^th^, 10^th^, 15^th^ and 21^th^ day postvaccination for identification of interleukin 6 (IL6), interferon gamma (IFN γ) genes by real-time polymerase chain reaction (PCR) and measurement of phagocytic index. Serum samples were collected weekly till 15 weeks postvaccination for detection of serum Abs by HI test. Tracheal swabs from groups that challenged with H9N2 virus were collected and tested for virus shedding by real-time PCR.

## Results

### IFN γ response of vaccinated chickens

**Figure-1:**
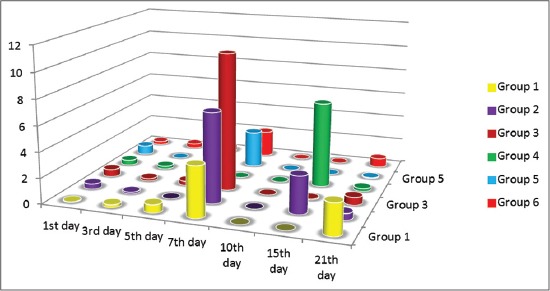
Interferon gamma response in chicken vaccinated with the prepared bivalent Newcastle disease virus and Avian influenza virus H9N2 at interval days postvaccination as measured by quantitative real-time reverse transcription - polymerase chain reaction assay.

Results of quantitative reverse transcription (qRT-PCR) assay to measure the IFN γ response of vaccinated chicken groups showed variability. Comparing routes of administration of the IMS1313, intranasal route induced response at 7 and 21, whereas spray route induced high response at 7, 15 and 21 days postvaccination. On the other hand, chicken group received Gel 01 via intranasal showed highest response only at 7^th^ compared to those administrated via spray which has response at 15^th^ day postvaccination (Figure-1).

### IL6 response of vaccinated chickens

**Figure-2:**
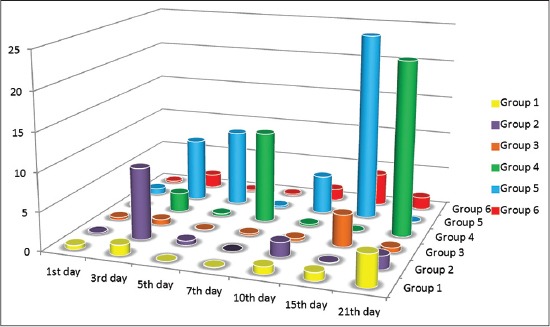
Interleukin 6 response in chicken vaccinated with the prepared bivalent Newcastle disease virus and Avian influenza virus H9N2 at interval days postvaccination as measured by quantitative real-time reverse transcription - polymerase chain reaction assay.

IL6 response of vaccinated groups of chicks at interval days as measured by qRT-PCR assay showed waves of increasing and decreasing values differ from group to group as shown in Figure-2. Groups 2 and 4 showed discrepancies in the IL6 response with the highest response at 21^st^ days in Group 4. IL6 response in Groups 1 and 3 was low and insignificant. Chicken received Gel 01 via spray was the highest in their IL6 response. On the other hand, the chickens received the ISA71 showed gradual IL6 response at 3^rd^, 5^th^, 10^th^ day and reached the highest response at 15 days postvaccination (Figure-2).

### Phagocytic activities

Phagocytic percentage and index measured at interval day’s postvaccination by equations:

Phagocytic percentage = (Number of phagocytes which ingest *Candida*)/(total number of phagocytes) × 100

Phagocytic index = (Total numebr of phagocytes which ingest more than two *Candida*)/(total number of phagocytes which ingest *Candida*)

**Figure-3:**
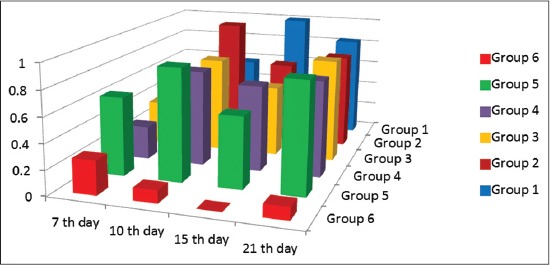
Phagocytic indices of vaccinated chickens at interval days as measured by phagocytic activity test.

The results showed gradual enhancement and the highest activity was in the 7^th^ and 21^th^ day for all groups except Group 1 where increase in activity was at the 15^th^ day and 21^th^ (Figure-3).

### Serological response

**Figure-4:**
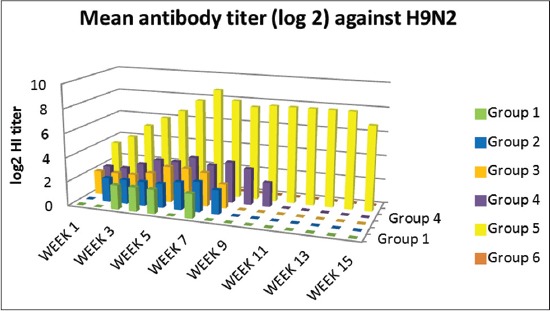
Hemagglutination inhibition means antibodies titer for H9N2 virus in sera of vaccinated chickens.

Serological response for H9N2 and NDV of vaccinated chicken was monitored by HI test for 15 weeks. H9N2 Abs were detected in a low titer which was not increased above 5 log 2 in vaccinated Groups 1-4, Group 5 has a higher titer which reached to 9 log 2 at the 7^th^ till 15^th^ week postvaccination (Figure-4).

**Figure-5:**
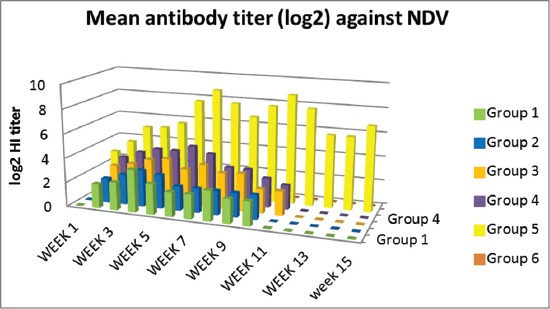
Hemagglutination inhibition means antibodies titer for Newcastle disease virus in sera of vaccinated chickens.

HI titers for NDV in sera of vaccinated chickens in different groups received the prepared mucosal vaccine increased and persisted till 7-9 weeks postvaccination except group 4 which received Gel 01 vaccine spray showed titers till 11 weeks. On the other hand, the ISA71 vaccinated group demonstrated high HI titers (with a peak of 8 log 2 at 8 weeks) persist till the 15 weeks postvaccination (Figure-5).

### Challenge trial

#### Protection %

**Figure-6:**
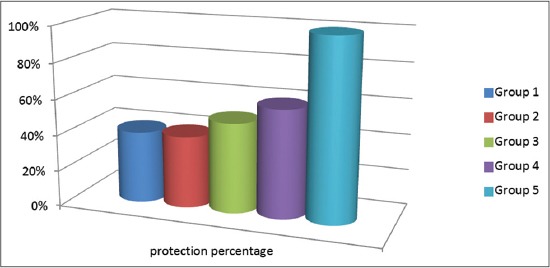
Protection % against the velogenic Newcastle disease virus genotype VIId of different vaccinated and challenged chickens.

After challenge with velogenic NDV genotype VIId NDV, the protection % for groups received IMS1313 vaccine was 40%. Groups received the Gel 01 vaccine revealed 50% and 60% for intranasal and spray route; respectively. On the other hand, the chickens vaccinated with ISA71 vaccine demonstrated 100% protection (Figure-6). All chickens in positive control group were dead within 48 h after challenge.

#### Shedding ratio after challenge with H9N2 virus

The shedding in trachea after challenge with H9N2 for Groups 1-5 and positive control group at interval days measured by real-time RT-PCR. No shedding was detected in samples from Groups 4 and 5 (received Gel 01 and ISA71 vaccines, respectively) in all interval days. Groups 1 and 2 showed one log reduction in shedding compare to the positive control group, whereas Group 3 showed 4, 3, and 0 logs reduction at 2, 4 and 6 days post challenge, all groups showed no mortalities but showed flu-like signs (Table-1).

**Table-1:** The shedding ratio after challenge with H9N2.

Groups	2 days post challenge			4 days post challenge			6 days post challenge		
		
	Result	Ct	Shedding amount (EID_50_)	Result	Ct	Shedding amount (EID_50_)	Result	Ct	Shedding amount (EID_50_)
IMS/N	Negative	No Ct	-	Positive	22.91	6.242×10^6^	Positive	25.18	1.277×10^6^
IMS/s	Positive	29.01	8.778×10^4^	Positive	24.24	2.463×10^6^	Positive	26.77	4.202×10^5^
GEL/N	Positive	35.53	9.303×10^2^	Positive	30.14	3.984×10^4^	Negative	No Ct	-
GEL/S	Negative	No Ct	-	Negative	No Ct	-	Negative	No Ct	-
ISA 71	Negative	No Ct	-	Negative	No Ct	-	Negative	No Ct	-
Control (+ve)	positive	20.34	3.534×10^7^	positive	21.9	1.256×10^7^	positive	23.92	3.081×10^6^

EID_50_=Embryo infective dose 50%

## Discussion

Mucosal vaccination could be a very useful solution for efficient and protective vaccination of poultry flocks [37]. Development of mucosal delivered vaccines may induce superior protection against viruses that infect via mucosal surfaces by stimulating the cellular immunity. This study documents the immunoenhancing effects of montanide adjuvants on immune response of vaccinated birds. In this regard, we prepared two new mucosal bivalent vaccines for NDV and AIV H9N2 with nanoparticles and polymer adjuvants. The nanoparticles and polymers vaccine adjuvant can enhance the efficacy of avian mucosal vaccination against infectious diseases [38, 39]. We used qPCR to characterize the expression of IFN γ and IL6 genes to provide insights into the role of innate immune response in protection against NDV and AIV H9N2 infection. The IMS1313 intranasal route induced response at 7 and 21 whereas spray route induced a high response at 7, 15 and 21 days postvaccination. On the other hand, chicken group received Gel 01 via intranasal showed the highest response only at 7^th^ compared to those administrated via spray which has response at 15^th^ day postvaccination, these polymers have the ability to activate cellular immune response in the host [40] possibly, polymers may also interact and activate various toll-like receptors, thus involving several innate immune system players in immune response [41]. IFNs are important members of host innate arm of immunity to prevent virus infection by their antiviral effect and triggering apoptosis [42, 43]. IL6 response of vaccinated groups of chicks showed waves of increasing and decreasing values differ from group to group. Chicken received Gel 01 via spray was the highest in their IL6 response. This increase in IL6 has a variety of functions, including the induction of acute phase proteins which impacts metabolic pathways [43], macrophage activation [44]. Phagocytic percentage and index measured at interval day’s postvaccination showed gradual enhancement and the highest activity was in the 7^th^ and 21^th^ day for all groups. This increase as a result of enhancing in IL6 and IFN γ as previously reported by Hu *et al*. [42], Katze *et al*. [43], Lienenluke and Christ [44]. H9N2 and NDV Abs were detected in low titers which were not increased above 5 log 2 in mucosaly vaccinated groups. These results were approved by others [45-47] who granted that aerosolized vaccines did enter the chicken respiratory tract either that’s why the results showed low Abs titer and they suggested that the dose of influenza virus used was insufficient or a booster vaccination would be needed in order to induce detectable levels of Ab, in the other hand the protection % was determined after 3 weeks postvaccination in vaccinated groups, the protection % of the polymer based adjuvant and ISA 71 were the most adjuvant formulations able to confer protection at high rate against infection with VVNDV and with no shedding in oropharyngeal swabs against infection with H9N2 AIV, these results confer to the Gel 01 formulation with spray route which is more efficient than the nanoparticles formulation and can enhance the efficacy of mass spray delivery of inactivated viral vaccines. This could be attributed to the mucoadhesive nature of the polymer compounds which give more contact time with respiratory tract mucosa hence give the chance to the innate immune response to take their role in protecting immune response [48-51]. It is known that that adaptive immune response initiated and regulated with previous non-specific innate immune response. There is marked elevation of IL6 gene expression early after vaccination which is very important for induction and regulation of innate immune response and directed the immune response to cellular immune response [38] Montanide gel 01 has been demonstrated to induce strong infiltration of monocytes and macrophages, so the inhanced phagocytosis of the antigen complex with the polymer increasing the activity of antigen-presenting cells, therefore, the innate immune response will trigger the adaptive immune system to induce a highly specific immune response [48,52,53]. The benefits anticipated from the use of adjuvant in inactivated vaccines concern both safety of the vaccine as the possible adverse reactions observed after delivery of live infectious vaccines could be lowered. Moreover, the risk of reversion to virulence that has already been observed in avian species could be also reduced [54,55]. The use of vaccines adjuvants expected to reduce the number of not responding birds and therefore reduce the possible reservoir for the infectious diseases [56].

## Conclusion

Our work underlines the ability to use polymer adjuvant in mass vaccination for avian species against viral infections, opening doors to improvements of inactivated avian vaccines safety and efficacy.

## Authors’ Contributions

This work was a part of PhD thesis of HEM supervised by HAH and MSM. HME: Conducted the laboratory animal experimental work and drafted and revised the manuscript. MSM: Shared in design of the experimental work and follow up the practical part of the research. HAH: Sets the design, supervised the work, drafted and revised the manuscript. All authors have read and approved the final manuscript.
